# Correlation Analysis of CXCL10, FOS, HOXC13, and WNT4 Gene Polymorphisms with Key Economic Traits—Initial Population Screening for Jiangnan Cashmere Goats

**DOI:** 10.3390/genes16091097

**Published:** 2025-09-16

**Authors:** Gvlnigar Amar, Qingwei Lu, Asma Anwar, Sen Tang, Qingfa Yan, Cuiling Wu, Xuefeng Fu

**Affiliations:** 1 Xinjiang Key Laboratory of Special Species Conservation and Regulatory Biology, College of Life Sciences, Xinjiang Normal University, Urumqi 830017, China; 2 Xinjiang Key Laboratory of Animal Biotechnology, Key Laboratory of Genetic Breeding and Reproduction of Herbivorous Livestock of Ministry of Agriculture and Rural Affairs, Xinjiang Uygur Autonomous Region Academy of Animal Science, Urumqi 830011, China; lqw120418@163.com (Q.L.);

**Keywords:** Jiangnan cashmere goat, key economic traits, SNPs, correlation analysis

## Abstract

**Background/Objectives**: The Jiangnan cashmere goat is a newly developed national cashmere goat breed in China, and the genetic stability of its traits is the core of breeding work. **Methods**: This study used 353 Jiangnan cashmere goats as research subjects. Descriptive statistics were performed on the key economic traits of the experimental population. Polymorphisms in the *CXCL10*, *FOS*, *HOXC13*, and *WNT4* genes were detected using multiplex PCR. The correlation between single-nucleotide polymorphism (SNP) loci and key economic traits was analyzed using the least squares variance method in SAS 9.4 software. **Results**: A total of 14 SNP loci were detected in the four genes, of which 5 were in the *CXCL10* gene. Three SNPs were detected in the *FOS*, *HOXC13*, and *WNT4* genes. SNP3, SNP4, SNP6, SNP10, SNP11, SNP12, SNP13, and SNP14 were in Hardy–Weinberg equilibrium. The results of the correlation analysis showed that SNP9 of the *HOXC13* gene was significantly correlated with birth weight (BW) and mean fiber diameter (MFD), SNP10 of the *HOXC13* gene was significantly correlated with yearling weight (YW), and SNP14 of the *WNT4* gene was significantly correlated with birth weight (BW) (*p* < 0.05). **Conclusions**: The results of this study provide molecular markers for cashmere goat breeding and an experimental basis for accelerating the cultivation of new strains, which is conducive to further optimizing the economic traits of the Jiangnan cashmere goat and ensuring the stable inheritance of its economic traits through molecular breeding.

## 1. Introduction

China is one of the core countries in the global cashmere industry. Both the number of cashmere goats raised and cashmere production in China have ranked first in the world for many years. Its industrial development is deeply integrated into multiple dimensions of increasing farmers’ income, regional economic growth, and ecological protection. With the continuous improvement in China’s national living standards, the market demand for high-quality cashmere products is growing, and high-end cashmere products are receiving increasing attention and demand. The technical system of the cashmere goat and sheep industry is also continuously advancing the breeding of this variety. As the production performance of the array continues to improve, the level of innovation and utilization of excellent germplasm resources is gradually rising [1].

The Jiangnan cashmere goat is a new breed of cashmere goat successfully developed in China through over 30 years of systematic breeding. It was created by using Liaoning cashmere goats as the sire and local Xinjiang goats as the dam, resulting in a breed with excellent cashmere production and strong adaptability. This breed features an all-white coat, high fleece yield, stable genetics, and strong stress resistance, enabling it to thrive in the extreme rearing conditions of southern Xinjiang [2]. It is primarily distributed in typical pastoral areas of southern Xinjiang, such as Aksu [3]. Under the influence of long-term drought, cold, sparse vegetation, natural environment, and artificial breeding, the Jiangnan cashmere goat gradually formed the characteristics of strong adaptability, roughage resistance, and excellent production performance, especially adapted to the ecological conditions of the gobi desert. It is suitable for the breeding habits and actual production conditions of farmers and herdsmen in southern Xinjiang. Our research group initially identified multiple key candidate genes (including *CXCL10*, *FOS*, *HOXC13*, and *WNT4*) related to fibrous and growth traits in sheep and goats based on genomic, transcriptomic, and proteomic analyses [4,5,6,7]. Whether these genetic polymorphisms can be used as genetic markers for molecular breeding of cashmere goats is unknown.

The protein CXCL10 can be synthesized and secreted by a variety of cell types, including fibroblasts, dendritic cells, and adipocytes [8]. CXCL10 is closely associated with the molecular mechanisms underlying economic traits, including growth, development, and cashmere quality. In addition, the study found that the *CXCL10* gene can not only regulate insulin secretion but also inhibit muscle cell proliferation and intramuscular angiogenesis and act as an important hub gene in the signal network related to muscle metabolism [9,10,11]. In a previous study, the team identified several candidate genes closely related to cashmere fineness through comprehensive analysis at the transcriptome and proteome levels. Based on GO and KEGG pathway enrichment analysis, it was found that these genes were significantly enriched in signaling pathways such as Notch, including 14 differentially expressed genes, especially *CXCL10* [7]. The *FOS* gene is a member of the *FOS* gene family, which is part of the AP-1 regulatory factor and plays a key role in the regulation of cell function. It has been proposed that *FOS* and its family member *FOSB* may affect the physiological characteristics of goat muscle fibers and the proliferation and differentiation of keratinocytes by regulating the activity of intracellular Ca^2+^ channels [12]. In addition, androgenetic alopecia studies have shown that platelet lysates can significantly inhibit the expression level of *FOS*. This indicates that *FOS* may play an important role in the regulation of epidermal inflammation [13,14]. Although the biological function of the *FOS* gene has been studied, the specific mechanism of action in the development of hair follicles in cashmere goats is still unclear and needs to be further explored. The *HOXC13* gene belongs to the homeobox gene family. It has been found that there are significant differences in the expression levels of *Hox* genes at different stages of hair follicle morphogenesis, indicating that the *HOXC13* gene may be involved in the regulation of hair follicle morphogenesis [13,14]. In addition, the *Hox* gene family may regulate the expression of hair keratin-specific genes and affect hair growth and development. Studies have shown that the *HOXC13* gene plays an important regulatory role in the occurrence and periodic growth of mouse hair [15]. In addition, the *HOXC13* gene promotes the transition of hair from the growth phase to the degenerative phase by inhibiting the TGF-βI signaling pathway, thereby affecting its growth process [16]. It can be seen that the *HOXC13* gene plays a key role in regulating hair growth and hair follicle development. Wnt is a secreted protein that activates some intracellular signaling pathways by binding to specific receptors on the cell membrane, thereby regulating a variety of life activities. Among these receptors, the Frizzled family is one of the core components of the Wnt signaling pathway [17]. As a key growth factor in this signaling network, Wnt4 shows high expression activity in mouse epidermal tissues and hair follicles. Related studies have shown that Wnt4 plays a key role in the regulation of hair follicle development and the formation of hair shaft structure by mediating the release of growth factors such as EGF and FGF during lysis [18].

Therefore, through gene polymorphism detection and correlation analysis, we explored the potential mechanisms of four candidate genes (*CXCL10*, *FOS*, *HOXC13*, and *WNT4*) in individual growth and development, hair follicle development, and hair fiber growth. The research results lay a theoretical foundation for molecular marker-assisted selection breeding of Jiangnan cashmere goats.

## 2. Materials and Methods

### 2.1. Selection of Experimental Animals

A total of 353 two-year-old female (non-lactating period) Jiangnan cashmere goats were selected from the Wenxiu County Sheep Breeding Center in Aksu Prefecture, Xinjiang Uygur Autonomous Region, China. All animal experiments were conducted in strict accordance with the guidelines established by the Animal Care and Use Committee of the Xinjiang Academy of Animal Science and Technology (Approval No. 2019009; Approval Date: 4 March 2019). Sample collection was performed under authorized conditions and complied with the Guidelines for the Care and Use of Laboratory Animals in China. All experimental goats were raised under identical feeding and management conditions. Missing values in the raw data were identified and excluded. Extreme or abnormal data were removed based on practical production conditions. The filtered data were used for subsequent analysis.

### 2.2. Collection and Measurement of Phenotypic Data

Based on the farm’s lambing, identification, and cashmere harvesting records, phenotypic data on these goats were gathered and organized. These data included birth weight (BW), yearling weight (YW), fleece weight (FW), and post-defleecing weight (PDW). A 10 g sample of cashmere fiber was collected from a point located 10 cm above the left center line of the shoulder blade for each individual. The mean fiber diameter (MFD), the fiber diameter standard deviation (FDSD), and the fiber diameter variation coefficient (CVFD) were assessed by utilizing the OFDA2000 instrument (BSC Electronics, Ardross, Australia). These measurements were used for subsequent analysis.

### 2.3. Sample Collection

We collected 5 mL of venous blood samples from the experimental goats, placed them in anticoagulant tubes, and stored them at −20 °C for subsequent experiments. We extracted DNA from the blood using a blood genomic DNA extraction kit (TIANGEN, Rockville, MD, USA). We used 1.0% agarose gel electrophoresis to detect DNA quality and determined DNA concentration to ensure sample integrity and experimental reliability [4].

### 2.4. SNP Genotyping

Based on the sequences published by the NCBI for goats, the exon regions of *CXCL10* (Gene ID: 100860873), *FOS* (Gene ID: 102171520), *HOXC13* (Gene ID: 102178245), and *WNT4* (Gene ID:102187819) genes were selected as the research targets. A primer pool covering the exon regions of four target genes was designed using Primer 5.0 and synthesized by Sangon Biotech (Shanghai) Co., Ltd. (Shanghai, China).Primer information is shown in Table 1. Subsequently, a BIO-RAD T100 TM PCR instrument was used to amplify the target SNP locus sequence via a two-step PCR method, and a library compatible with Illumina sequencing was constructed. Finally, the HiSeq XTen sequencer (Illumina, San Diego, CA, USA) was used for high-throughput sequencing to ensure the accuracy and integrity of the data [19].

### 2.5. Quality Control of Sequencing Data

Cutadapt software (Version 1.2.1) was used to remove the adaptor sequence in the sequencing data, and the first 10 bases were removed from the 5′ and 3′ ends of the reads. Subsequently, PRINSEQ-lite (Version 0.20.3) was used for data quality control. According to the 3′ → 5′ direction, low-quality bases with a Phred quality value < 20 were removed to improve the reliability of sequencing data.

### 2.6. Statistical Analysis

Popgene software (Version 1.32) was used to determine the genetic polymorphism statistics of SNP loci, and the SNP minor allele frequency (MAF) and Hardy–Weinberg equilibrium (HWE) were calculated. Combined with the results of key economic traits of Jiangnan cashmere goats, the correlation between SNP genotype and traits was systematically analyzed by using the GLM method in SAS 9.4. The results are expressed in the form of least squares mean ± standard error, and the linear model is as follows:

Y_ick_ = G_i_ + H_c_ + e_ick_ (1)

In the formula, Y_ick_ is the individual phenotypic value of a cashmere goat; G_i_ is the genotype SNP effect; H_c_ is the field effect; and e_ick_ is the random error.

## 3. Results

### 3.1. Descriptive Statistics on Key Economic Traits

Descriptive statistical analysis was carried out on the BW, YW, FW, PDW, MFD, FDSD, and CVFD of Jiangnan cashmere goats. After excluding outliers, the effective sample size (N) varied due to differences in data completeness across indicators. The sample data are presented in Table 2. Among them, the sample size for FDSD and CVFD was large, at 353 individuals. The sample size for FW was the smallest, at 344 individuals. The data obtained from the analysis results for each trait were within the normal range; among these, the standard deviation (SD) for YW was 3.53, indicating a certain degree of fluctuation around the mean. The coefficients of variation for BW (CV = 28.10%) and FW (CV = 24.71%) were relatively high, suggesting significant individual variation within the population for these traits and indicating substantial potential for genetic improvement. Additionally, the coefficients of variation for MFD, PDW, FDSD, and CVFD ranged between 7% and 14%, indicating moderate variability. This suggests these traits exhibit strong stability within the population.

### 3.2. Annotation of Typing Results

Using multiplex PCR and exome sequencing, 14 mutation sites were identified in four genes (*CXCL10*, *FOS*, *HOXC13*, and *WNT4*) in the Jiangnan cashmere goat. These sites included five sites in the *CXCL10* gene (SNP1, SNP2, SNP3, SNP4, and SNP5), three sites in the *FOS* gene (SNP6, SNP7, and SNP8), three loci in the *HOXC13* gene (SNP9, SNP10, and SNP11), and three loci in the *WNT4* gene (SNP12, SNP13, and SNP14). Except for the last site (SNP14), which is a synonymous mutation, all other sites are non-coding region mutations (SNP1-SNP13), including one insertion mutation (SNP7). The results are shown in Table 3.

### 3.3. Analysis of Population Genetic Polymorphism

A population genetic polymorphism analysis was conducted on 14 mutation sites in the Jiangnan cashmere goat population. The results are shown in Table 4; a total of five genotypes were identified across the 14 sites, including four A, G types (AA, AG, GG; including SNP1, SNP4, SNP8, SNP10), seven C, T types (CC, CT, TT; including SNP2, SNP3, SNP5, SNP11, SNP12, SNP13, and SNP14), one T, G type (TT, TG, GG; SNP6), one A, T type (AA, AT, TT; SNP9), and one A, Ains_A type (SNP7). In addition, the results show that eight loci exhibited polymorphism (He > 0.2), among which seven loci (SNP1, SNP2, SNP3, SNP7, SNP8, SNP9, and SNP11) exhibited moderate polymorphism (0.5 > He > 0.3) and one locus (SNP14) exhibited high polymorphism (He = 0.5). The Hardy–Weinberg equilibrium test results show that eight SNP loci were in Hardy–Weinberg equilibrium (*p* > 0.05), including SNP3, SNP4, SNP6, SNP10, SNP11, SNP12, SNP13, and SNP14, while six loci were not in Hardy–Weinberg equilibrium (*p* < 0.05), including SNP1, SNP2, SNP5, SNP7, SNP8, and SNP9.

### 3.4. The Significance Analysis of SNPs and Key Economic Traits

Four SNPs (SNP5, SNP6, SNP12, and SNP13) with a minor allele frequency (MAF < 0.05) were excluded. The correlation between the remaining ten SNP loci of the four genes and the key economic traits was analyzed (Table 5).

The results show that SNP9 of the *HOXC13* gene had a significant effect on BW and MFD (*p* < 0.05) and SNP10 of the *HOXC13* gene had a significant effect on YW (*p* < 0.05). SNP14 of the *WNT4* gene had a significant effect on BW (*p* < 0.05). It is worth noting that although the genotypes of the remaining SNPs did not show significant or extremely significant effects on the traits, the values of individual traits in different genotypes of the same SNPs were different. For example, the BW level of homozygous genotypes in SNP1, SNP2, SNP3, and SNP14 was higher than that of heterozygous genotypes. The MFD level of SNP1 showed an increasing trend in AA, AG, and GG, while it showed a decreasing trend in SNP2. It can be seen that the genotype has a certain effect on these phenotypes.

## 4. Discussion

With the wide application of molecular markers in multiple research and production fields, they have become an indispensable part of molecular genetics, greatly promoting research on the genetic basis of economic traits in sheep. Compared with traditional breeding methods, molecular genetic technologies show obvious advantages in selection efficiency and genetic stability, and are gradually becoming an important supporting tool in modern breeding systems. Genome-wide, DNA sequence variations caused by single-nucleotide mutations are called SNPs. SNPs can be distributed in any region of the genome and widely exist in the gene sequences of different species [20]. In essence, they refer to DNA sequence polymorphisms caused by substitutions, insertions, or deletions of single bases in different alleles at the same locus [21]. The application of molecular marker technology in sheep breeding can effectively eliminate the influence of age, gender, and environmental conditions on trait determination and improve the efficiency and accuracy of individual identification, thus promoting the optimization of population structure and the expansion of excellent genetic resources [22]. Therefore, screening genes and molecular markers related to key economic traits is of great scientific value and practical significance for the genetic improvement and efficient breeding of Jiangnan cashmere goats.

### 4.1. Association Analysis Between CXCL10 Gene SNPs and Key Economic Traits

CXCL10, also known as interferon-induced protein 10 (IP-10), is a member of the CXC chemokine family. It contains four exons and three introns [23]. As a newly discovered muscle growth regulatory factor, *CXCL10* is involved in multiple physiological processes, including the regulation of insulin secretion, the inhibition of myocyte proliferation, and intramuscular angiogenesis. It is a key regulatory gene in the muscle metabolic network and is closely related to individual growth and development [9,23]. Studies have shown that *CXCL10* is at the core of the muscle development regulatory network, and its mutations may change the functional performance and transcriptional activity of the gene by affecting the binding sites of transcription factors [24]. It is worth noting that *CXCL10* has also been found to be involved in the regulation of hair follicle development. Analysis found that the *CXCL10* gene is closely related to the formation of MFD [25]. There are also findings that there is a significant correlation between the loss of scalp and body hair and inflammatory reactions, in which chemokines and their receptors play an important role in the pathogenesis of Alopecia Areata (AA) [26,27]. Studies have shown that *CXCL10* is highly expressed in AA patients, which may induce hair loss by promoting the aggregation of T cells to inflammatory sites. This phenomenon may be related to the natural shedding mechanism of secondary hair follicles in cashmere goats during the resting period. It has also been found that *CXCL10* is a specific transcriptional target of Ectodysplasin-mediated hair growth, indicating that it may play an important regulatory role in cashmere regeneration or the formation and development of secondary hair follicles [28]. In addition, Chen Zhi et al. took Qianbei Ma goats, Inner Mongolia cashmere goats, and Kanto dairy goats as research objects, and used DNA pool construction and sequencing technology to identify two SNPs in the coding region of the *CXCL10* gene that may be related to reproductive traits [29].

The *CXCL10* gene has important functions in muscle growth, immune inflammation, hair follicle development, and reproduction. In this study, although the BW levels fluctuated among different genotypes of *CXCL10*, there was no significant difference. This may be because the function of the *CXCL10* gene is affected by a variety of regulatory mechanisms, and its SNPs may be located in non-critical regions or have weak effects. In addition, external factors such as breeding environment, feeding management, and nutritional level may affect the expression level of *CXCL10*, thus failing to show a significant impact on the corresponding traits. Therefore, further research still needs to combine larger sample sizes and multi-omics analysis to deeply explore the mechanism of action of the *CXCL10* gene in economic traits.

### 4.2. Association Analysis Between FOS Gene SNPs and Key Economic Traits

The *FOS* gene belongs to the catalytic protein 1 family and plays an important role in regulating cell growth, division, and proliferation, as well as in the animal immune system [26]. Early studies found that *FOS* affects the structure of hair follicle cell membranes and the regulation of enzyme activity, and may play a role in hair follicle keratinization and the proliferation and differentiation of secondary hair follicle cashmere, thereby regulating the cashmere shedding cycle of cashmere goats [30]. In addition, *FOS* shows a similar expression pattern in normal keratinized tissues (such as epidermis and oral epithelium) and shows positive expression in the early stage of keratinization, suggesting that it plays an important role in regulating the expression of keratinization-related genes [31,32]. In the transcriptomic analysis of Changthangi goats, the *FOS* gene was proposed to play a role in goat hair development and growth, thereby influencing fleece production [33]. In addition, *FOS* is widely involved in intracellular signal transduction and energy metabolism, and plays an important role in cell proliferation, differentiation, and stress response [34]. Although the *FOS* gene has important biological functions in cell proliferation, differentiation, and hair follicle development, in this study, the three mutation sites of the *FOS* gene did not show a significant impact on key economic traits. Future studies will further investigate the impact of this gene on hair follicle development and cashmere growth at the transcriptome level.

### 4.3. Association Analysis Between HOXC13 Gene SNPs and Key Economic Traits

The *HOXC13* gene belongs to the homeobox (*Hox*) gene family and plays a key role in cell differentiation, tissue-specific gene expression, and embryonic development [35,36,37,38,39]. *HOXC13* affects the formation and development of hair follicles mainly by regulating keratin (KP) and its related proteins (KAP), and determines the expression of hair keratin-specific genes [40,41,42]. *HOXC13* is the first *Hox* gene proven to play a key role in hair growth and development. When the gene is deleted or overexpressed, mice show a hairless phenotype, indicating its core role in hair follicle development. In addition, *HOXC13* regulates the transition of the hair cycle by inhibiting the TGF-βI signaling pathway, thereby affecting hair growth [16]. In the study of sheep hair follicles, the expression level of *HOXC13* varies significantly in different periods of the hair follicle cycle, indicating that it plays an important regulatory role in hair follicle morphogenesis [15]. *HOXC13* is highly conserved, and its deletions and synonymous and missense mutations may lead to diseases such as pure hair–nail ectodermal dysplasia (PHNED), thereby affecting hair growth [43,44,45]. In Shanbei white cashmere goats and Inner Mongolia white cashmere goats, no mutations related to the *KAP16.2* and *KAP16.6* genes were detected in the *HOXC13* gene, further confirming the high genetic conservation of the *HOXC13* gene in these breeds [46]. Although no polymorphism of this gene was found in the above two breeds, the CDS sequence of the *HOXC13* gene was successfully obtained in Tibetan cashmere goats, and it was revealed that it has obvious polymorphism and specific expression patterns [47]. The study further found that the SNP site in the *HOXC13* gene is significantly associated with the fiber diameter trait of Tibetan cashmere goats, indicating that the *HOXC13* gene has the potential to be used as a molecular marker in ultra-fine cashmere breeding [25]. Research on hair follicle development in Shanbei white cashmere goats indicates that *HOXC13* plays a crucial role in processes such as hair shaft differentiation and follicular keratinization. Furthermore, findings from the Changthangi goat study support the notion that the *HOXC13* gene influences keratin formation and follicular development. Comparisons with prior mouse and human-related results further validate the reliability and authenticity of this gene’s association with animal hair growth [33,48]. Furthermore, research on human hair has indicated that *HOXC13* participates in regulating the expression of hair keratin during the early differentiation of hair follicle cells. Additionally, studies have linked *HOCX13* to hereditary immunodeficiency with alopecia and localized autosomal recessive hypohair syndrome [40,49]. In addition, studies have found that the methylation level of CpG sites in the promoter region of the *HOXC13* gene is significantly higher in the cashmere resting period than in the growing period, indicating that epigenetic modification of the *HOXC13* gene may be involved in the regulation of the hair follicle growth cycle [50].

This study found that the SNP9 site of the *HOXC13* gene had a significant impact on BW and MFD, SNP10 of the *HOXC13* gene had a significant impact on the YW of Jiangnan cashmere goats, and individuals carrying the AT genotype showed a higher BW level compared with those with the AA genotype of the SNP9. This result indicates that the genetic variation at the SNP9 site may play a key role in regulating the early growth and development of individuals. Therefore, the *HOXC13* gene and its SNP9 and SNP10 sites have the potential to be used as molecular breeding targets, providing a theoretical basis and technical support for future molecular design breeding in Jiangnan cashmere goats. In addition, the minor allele frequency (MAF) of SNP9 is low, which suggests that future studies should expand the sample size to improve statistical power and further verify the genetic effect of this site. At the same time, this also reflects the genetic diversity of this gene locus in the Jiangnan cashmere goat population, which may be related to the genetic background of different cashmere goat breeds.

### 4.4. Association Analysis Between WNT4 Gene SNPs and Key Economic Traits

As a key member of the Wnt signaling pathway, *WNT4* is considered one of the core growth regulatory factors and plays an important role in the development of the kidney, urinary system, adrenal gland, mammary gland, pituitary gland, and female reproductive organs [51,52]. Wnt4 is also a key ligand in the Wnt pathway and has significant regulatory functions in the maturation of mammalian ovarian granulosa cells and hair follicle formation [53]. The *WNT4* gene is actively expressed in mouse epidermis and hair follicles, and can regulate the release of a series of factors related to hair follicle development, such as epidermal growth factor and fibroblast growth factor, thereby having an important impact on the normal growth of cashmere and the formation of hair shaft fibers [18]. The research team earlier conducted an association analysis on the SNP sites of *WNT4* and *HOXC13* genes in Tibetan cashmere goats, and the results showed that each of the two genes had two SNP sites significantly correlated with cashmere fineness traits (*p* < 0.05) [25]. In this study, the SNP site of *WNT4* did not show a significant impact on the traits of Jiangnan cashmere goats, but had a significant impact on BW, and individuals with the CC genotype had a significantly higher BW than those with the CT genotype. This result indicates that the genetic variation at the SNP14 site may play a key role in regulating the growth and development of goats. The MAF of SNP14 is high, indicating that this mutation has high genetic diversity in the population. It can have potential value for the selection of goat growth traits.

## 5. Conclusions

We conducted a correlation analysis of four genes (*CXCL10*, *FOS*, *HOXC13*, and *WNT4*) with seven key economic traits (BW, YW, FW, PDW, MFD, FDSD, and CVFD) in Jiangnan cashmere goats. The results showed that *HOXC13* SNP9 and *WNT4* SNP14 were significantly correlated with BW, SNP9 was also significantly correlated with MFD, and the SNP10 of the *HOXC13* gene was significantly correlated with YW. In practice, these results provide potential targets for the molecular-assisted selection of Jiangnan cashmere goats and serve as important genetic markers for phenotypic quality. They can also be used to improve the evaluation of genetic potential in terms of economic traits.

## Figures and Tables

**Table 1:** Primer information of four genes.

Gene Name	Length/bp	Primer
*CXCL10*	420	F: TTTGTCTTACATAGCCTGCAGAACA
		R: CTTCTTCCCCTTTCCAATCTTTCTA
*FOS*	432	F: ACACAGATCCCTTATGTCTGGTCTC
		R: TGATAGTCTCACCCTAAGAAATGCC
*HOXC13*	311	F: CACGACAGTGAAAACAAATTAGTGG
		R: CTCTAATCTTAGGTTCTTGAGGCCC
*WNT4*	382	F: ACAGCCACACTTCTCCAGCTC
		R: GAAAATGGGGATGACAGTTGTACTT

**Table 2:** Descriptive statistics of key economic traits in Jiangnan cashmere goats.

Trait	N	Min	Max	Mean	SD	CV
BW, kg	346	1.2	4.2	2.74	0.77	28.10
YW, kg	347	13	33	21.68	3.53	16.28
FW, g	344	160	675	411.84	101.76	24.71
PDW, kg	351	16	36	26.01	3.42	13.15
MFD, μm	351	11.7	18.9	15.67	1.17	7.46
FDSD	353	2.4	4	3.26	0.25	7.67
CVFD	353	16.9	25.2	20.85	1.44	-

**Table 3:** Mutation site information of four genes.

Gene	SNP	Position	Nucleotide Variation	Amino Acid Variation
*CXCL10*	SNP1	6:91399380	A → G	-
	SNP2	6:91399532	C → T	-
	SNP3	6:91399379	C → T	-
	SNP4	6:91399401	A → G	-
	SNP5	6:91399473	T → C	-
*FOS*	SNP6	10:16633885	G → T	-
	SNP7	10:16633856	A → ins_A	-
	SNP8	10:16633782	G → A	-
*HOXC13*	SNP9	5:25823773	A → T	-
	SNP10	5:25823481	G → A	-
	SNP11	5:25823671	C → T	-
*WNT4*	SNP12	2:5248863	C → T	-
	SNP13	2:5248500	C → T	-
	SNP14	2:5248554	T → C	S

**Table 4:** The basic characteristics of 14 single-nucleotide polymorphisms of four genes.

Gene	SNPs	Genotype	Freq	Allele_P	Allele_Q	He	Ne	HWE *p*-Value
*CXCL10*	SNP1	AA (129)	0.4510	0.5052	0.4948	0.4999	1.9998	0.0000
		AG (31)	0.4406					
		GG (126)	0.1084					
	SNP2	CC (200)	0.7168	0.7294	0.2706	0.3948	1.6522	0.0000
		CT (7)	0.2581					
		TT (72)	0.0251					
	SNP3	CC (180)	0.6383	0.7996	0.2004	0.3204	1.4715	0.9053
		CT (91)	0.0390					
		TT (11)	0.3227					
	SNP4	AA (266)	0.9301	0.9633	0.0367	0.0707	1.0761	0.3042
		AG (19)	0.0035					
		GG (1)	0.0664					
	SNP5	CC (1)	0.0031	0.0109	0.9891	0.0216	1.0220	0.0000
		CT (5)	0.9813					
		TT (315)	0.0156					
*FOS*	SNP6	TT (0)	0.0000	0.0417	0.9583	0.0799	1.0868	0.7942
		GT (3)	0.9167					
		GG (33)	0.0833					
	SNP7	AA (260)	0.7365	0.7365	0.2635	0.3881	1.6342	0.0000
		Ains_A (93)	0.2635					
			0.0000					
	SNP8	AA (87)	0.2465	0.5340	0.4660	0.4977	1.9908	0.0035
		AG (203)	0.1785					
		GG (63)	0.5751					
*HOXC13*	SNP9	AA (205)	0.8613	0.8613	0.1387	0.2389	1.3138	0.0000
		AT (33)	0.0000					
		TT (0)	0.1387					
	SNP10	AA (0)	0.0000	0.0938	0.9062	0.1699	1.2047	0.0642
		AG (60)	0.8125					
		GG (260)	0.1875					
	SNP11	CC (146)	0.4136	0.6487	0.3513	0.4558	1.8374	0.5502
		CT (166)	0.1161					
		TT (41)	0.4703					
*WNT4*	SNP12	CC (335)	0.9824	0.9912	0.0088	0.0174	1.0178	0.8698
		CT (6)	0.0000					
		TT (0)	0.0176					
	SNP13	CC (301)	0.9495	0.9748	0.0252	0.0492	1.0517	0.6448
		CT (16)	0.0000					
		TT (0)	0.0505					
	SNP14	CC (87)	0.2465	0.5000	0.5000	0.5000	2.0000	0.7901
		CT (179)	0.2465					
		TT (87)	0.5071					

Note: Allele_P: major allele frequency; Allele_Q: minor allele frequency; He: expected heterozygosity; Ne: effective number of alleles. HWE *p*-value: Hardy–Weinberg equilibrium (*p* > 0.05 indicates conformity to equilibrium, *p* ≤ 0.05 indicates statistically significant deviation from HWE).

**Table 5:** Correlation analysis between genotypes of different SNP loci and key economic traits.

Gene	SNPs	Genotype	BW/kg	YW/kg	FW/g	PDW/kg	MFD/μm	FDSD	CVFD
*CXCL10*	SNP1	AA	2.72 ± 0.08	21.77 ± 0.4	427.07 ± 10.53	26.18 ± 0.35	15.65 ± 0.10	3.24 ± 0.02	20.78 ± 0.13
		AG	2.66 ± 0.08	21.20 ± 0.4	407.18 ± 10.53	26.12 ± 0.35	15.74 ± 0.10	3.28 ± 0.02	20.88 ± 0.13
		GG	2.84 ± 0.15	20.88 ± 0.81	391.52 ± 21.32	24.97 ± 0.71	15.91 ± 0.21	3.30 ± 0.05	20.74 ± 0.26
	SNP2	CC	2.73 ± 0.06	21.44 ± 0.32	419.72 ± 8.46	25.90 ± 0.28	15.77 ± 0.08	3.28 ± 0.02	20.86 ± 0.10
		CT	2.70 ± 0.10	21.29 ± 0.53	402.17 ± 13.96	26.42 ± 0.46	15.66 ± 0.14	3.23 ± 0.03	20.70 ± 0.17
		TT	2.47 ± 0.33	22.64 ± 1.71	462.46 ± 44.84	26.82 ± 1.49	15.44 ± 0.44	3.22 ± 0.10	20.70 ± 0.54
	SNP3	CC	2.76 ± 0.06	21.61 ± 0.34	410.62 ± 8.94	26.32 ± 0.30	15.63 ± 0.09	3.25 ± 0.02	20.87 ± 0.11
		CT	2.59 ± 0.09	21.14 ± 0.47	428.81 ± 12.40	25.69 ± 0.41	15.87 ± 0.12	3.28 ± 0.02	20.77 ± 0.15
		TT	2.69 ± 0.26	19.94 ± 1.36	379.63 ± 35.75	23.99 ± 1.18	15.83 ± 0.35	3.27 ± 0.08	20.66 ± 0.43
	SNP4	AA	2.71 ± 0.05	21.37 ± 0.28	416.64 ± 7.36	25.97 ± 0.24	15.71 ± 0.07	3.26 ± 0.02	20.83 ± 0.09
		AG	2.66 ± 0.20	22.02 ± 1.04	379.95 ± 27.29	26.73 ± 0.90	15.72 ± 0.27	3.22 ± 0.06	20.52 ± 0.33
		GG	2.07 ± 0.86	24.03 ± 4.51	384.17 ± 118.34	31.39 ± 3.92	14.63 ± 1.18	3.39 ± 0.25	23.22 ± 1.42
*FOS*	SNP7	AA	2.67 ± 0.05	21.14 ± 0.28	414.63 ± 7.43	26.05 ± 0.25	15.64 ± 0.07	3.25 ± 0.02	20.83 ± 0.09
		Ains_A	2.79 ± 0.09	21.67 ± 0.47	415.66 ± 12.28	25.60 ± 0.41	15.71 ± 0.12	3.29 ± 0.03	21.00 ± 0.15
	SNP8	AA	2.83 ± 0.09	20.89 ± 0.48	436.33 ± 12.64	25.80 ± 0.42	15.70 ± 0.13	3.24 ± 0.03	20.71 ± 0.15
		GA	2.65 ± 0.06	21.55 ± 0.32	406.87 ± 8.34	25.73 ± 0.28	15.64 ± 0.08	3.26 ± 0.02	20.88 ± 0.10
		GG	2.62 ± 0.10	20.97 ± 0.57	411.00 ± 14.86	26.74 ± 0.49	15.64 ± 0.15	3.29 ± 0.03	21.07 ± 0.18
*HOXC13*	SNP9	AA	**2.59 ± 0.06 ^a^**	20.95 ± 0.31	412.13 ± 8.29	25.59 ± 0.27	**15.62 ± 0.08 ^a^**	3.24 ± 0.02	20.80 ± 0.10
		AT	**2.92 ± 0.15 ^b^**	20.98 ± 0.78	433.99 ± 20.54	26.98 ± 0.67	**16.06 ± 0.20 ^b^**	3.30 ± 0.04	20.70 ± 0.25
	SNP10	GG	2.64 ± 0.05	**20.94 ± 0.28 ^a^**	419.59 ± 7.41	26.91 ± 0.25	15.66 ± 0.07	3.26 ± 0.05	20.89 ± 0.26
		AG	2.80 ± 0.11	**22.33 ± 0.58 ^b^**	407.86 ± 15.21	25.54 ± 0.51	15.66 ± 0.15	3.26 ± 0.03	20.87 ± 0.18
	SNP11	CC	2.72 ± 0.07	21.46 ± 0.38	413.57 ± 9.87	26.11 ± 0.33	15.68 ± 0.10	3.26 ± 0.02	20.84 ± 0.12
		CT	2.67 ± 0.07	21.20 ± 0.35	419.03 ± 9.24	25.74 ± 0.31	15.69 ± 0.09	3.28 ± 0.02	20.93 ± 0.11
		TT	2.64 ± 0.13	20.98 ± 0.71	403.03 ± 18.49	26.06 ± 0.61	15.42 ± 0.18	3.19 ± 0.04	20.73 ± 0.22
*WNT4*	SNP14	CC	**2.87 ± 0.09 ^Aa^**	21.42 ± 0.48	425.08 ± 12.66	25.68 ± 0.42	15.69 ± 0.13	3.29 ± 0.03	21.00 ± 0.15
		CT	**2.58 ± 0.06 ^Bb^**	21.15 ± 0.34	415.53 ± 8.88	26.07 ± 0.30	15.62 ± 0.09	3.25 ± 0.02	20.88 ± 0.11
		TT	**2.72 ± 0.09 ^Aa^**	21.42 ± 0.49	403.02 ± 12.78	25.89 ± 0.43	15.69 ± 0.13	3.24 ± 0.03	20.70 ± 0.15

Note: If the data of the same column in the table are marked with different capital letters (such as A, B), the difference between the groups is extremely significant (*p* < 0.01); if different lowercase letters (such as a, b) are marked, the difference is significant (*p* < 0.05); unmarked letters or the same letters indicate that the difference is not statistically significant (*p* > 0.05). Significance is indicated by bold type.

## Data Availability

The original contributions presented in this study are included in the article. Further inquiries can be directed to the corresponding authors.

## Abbreviations

The following abbreviations are used in this manuscript:

BW	Birth Weight
YW	Yearling Weight
FW	Fleece Weight
PDW	Post-Defleecing Weight
MFD	Mean Fiber Diameter
FDSD	Fiber Diameter Standard Deviation
CVFD	Fiber Diameter Variation Coefficient
